# Overcoming Carbon-Shell Passivation in Biomass-Templated NiFe_2_O_4_/rGO@C Composites via a Urea-Assisted One-Pot Optimization Strategy for Enhanced Electrochemical Nitrite Sensing

**DOI:** 10.3390/molecules31172954

**Published:** 2026-08-23

**Authors:** Hanxu Liu, Khi Khim Beh, Jia Li, Wanling Lin, Chang Liu, Xin Liu, Chao Chen, Wenhao Chen, Mohamad Adzhar Md Zawawi

**Affiliations:** 1 School of Electrical and Electronic Engineering, Universiti Sains Malaysia, Nibong Tebal 14300, Penang, Malaysia; liuhanxu2021@gmail.com (H.L.);; 2 School of Physics and Electronic Engineering, Hanshan Normal University, Chaozhou 521041, China; 3 Collaborative Microelectronic Design Excellence Centre (CEDEC), Universiti Sains Malaysia, Nibong Tebal 14300, Penang, Malaysia; 4 School of Minerals and Energy Resources Engineering, University of New South Wales, Sydney, NSW 2052, Australia

**Keywords:** inverse spinel ferrite, carbon-shell passivation, biomass-templated carbonization, in-situ nitrogen doping, electrocatalytic nitrite detection, hierarchical pore engineering

## Abstract

Biomass-templated spinel ferrite/carbon nanocomposites are promising electrode materials; however, dense carbon shells formed during low-temperature carbonization can block electrolyte access to active sites and impair electrochemical performance. The role of this carbon-shell passivation in biomass-derived ferrite/carbon composites remains largely unexplored. Here, we identify this limitation in a stepwise-synthesized NiFe_2_O_4_/rGO@C composite (NFC-S, BET surface area = 5.23 m^2^ g^−1^, ΔEp = 113.3 mV) and resolve it through a rationally designed one-pot optimization strategy in which urea simultaneously serves as a pore-forming agent and nitrogen precursor. The optimized composite (N-NFC-O) achieves a BET surface area of 186.39 m^2^ g^−1^—a 35.6-fold enhancement—with 70.1% micropore contribution and 3.13 at.% in-situ nitrogen doping. Electrochemically, ΔEp narrows to 72.3 mV and enables efficient NO_2_^−^ oxidation with a ~70 mV cathodic shift, whereas NFC-S shows negligible catalytic response under identical conditions. As a proof of concept, differential pulse voltammetry (DPV) yields a nitrite sensitivity of 9.17 µA cm^−2^ mM^−1^ and a detection limit of 92.5 µM. Overall, this work identifies carbon pore accessibility as a key structural descriptor governing electrocatalytic performance in biomass-derived ferrite/carbon composites and provides a general design strategy for developing high-performance biomass-derived carbon/oxide hybrid electrodes.

## 1. Introduction

Nickel ferrite (NiFe_2_O_4_), an inverse spinel oxide composed entirely of earth-abundant elements, has attracted considerable attention as an electrocatalyst owing to its tunable mixed-valence redox couples (Fe^2+^/Fe^3+^, Ni^2+^/Ni^3+^) and thermodynamic stability in corrosive media [1,2,3,4]. Two intrinsic limitations, namely low electrical conductivity and nanoparticle aggregation, are commonly addressed by hybridization with conductive carbon scaffolds such as graphene/rGO or biomass-derived carbon [5,6,7,8]. Among various carbon supports, biomass-derived carbon is particularly attractive because it simultaneously serves as a structural template, carbon source, and heteroatom dopant through simple and scalable synthetic routes [6,9,10]. However, an important yet underexplored consequence of this strategy is that during moderate-temperature carbonization (400–600 °C), the carbon matrix can conformally encapsulate the oxide nanoparticles, restricting electrolyte accessibility and nullifying the catalytic benefit of the embedded active phase [11,12,13]. Nitrite is an important analytical target because of its widespread presence in environmental and food-related systems. Electrochemical sensing techniques offer rapid, highly sensitive, and cost-effective detection of essential analytes such as nitrite and hydrogen peroxide [14], however, sensing performance strongly depends on the accessibility of catalytic sites, interfacial electron transfer, and analyte diffusion. Therefore, electrode materials that simultaneously provide abundant electrochemically accessible active sites, continuous conductive pathways, and open porous structures are highly desirable. Forming composite materials by integrating transition metal oxides with conductive carbon matrices is a highly effective approach for enhancing overall electrode performance, as it resolves the inherent poor electronic conductivity of pristine oxides while preventing their aggregation during cycling [15]. In this context, spinel ferrite/carbon composites are particularly attractive because the oxide phase can provide redox-active sites, while the conductive carbon framework can facilitate charge transport and analyte diffusion.

In this work, we describe this phenomenon as “carbon-shell passivation”, referring to the paradoxical situation in which the conductive matrix intended to enhance performance instead acts as a diffusion barrier to the buried active sites. Although the detrimental effect of excessively thick carbon shells on electrocatalytic accessibility has been recognized in other systems [16,17,18], its manifestation in biomass-derived ferrite/carbon composites has received limited attention. Experimentally, this phenomenon is typically manifested by the BET surface area drops far below theoretical expectations (as low as 3–10 m^2^ g^−1^ for composites nominally containing rGO at ~2630 m^2^ g^−1^), cyclic-voltametric peak separations widen, and inner-sphere catalytic activity is suppressed—even when XRD confirms crystalline oxide phases [13,19]. The underlying mechanism is the incomplete pyrolysis of polysaccharide precursors, which generates a largely amorphous, non-porous carbon coating that seals interparticle voids [20]. Raising the pyrolysis temperature above 700 °C can partially alleviate the problem; however, excessively high temperatures may induce particle coarsening, carbon collapse, or carbothermal reduction of metal oxides [21]. The central question thus remains: how can the carbon-shell passivation barrier be disrupted without sacrificing the structural integrity of the embedded spinel ferrite?

Here we address this challenge using cotton cellulose fibers as a biomass template and urea (CO(NH_2_)_2_) as a multifunctional additive. Cotton provides a hierarchical architecture that templates multi-scale porosity upon carbonization [22,23,24], while its oxygen-rich backbone steers metal precursor oxidation toward thermodynamically stable spinel phases [25,26]. Urea undergoes thermal decomposition at 400–600 °C, releasing NH_3_, CO_2_, and HNCO [27,28] gases that generate abundant pores within the densifying carbon shell and simultaneously introduce nitrogen functionalities into the carbon framework (pyridinic-N, graphitic-N), a well-established promoter of electrocatalytic activity [10,29].

In this work, we systematically identify the origin of carbon-shell passivation in a stepwise-synthesized NiFe_2_O_4_/rGO@C composite (NFC-S, BET = 5.23 m^2^ g^−1^, ΔEp = 113.3 mV), then overcome through an integrated one-pot optimization strategy (N-NFC-O: 600 °C, Fe:Ni = 2:1, urea-assisted) that achieves a 35.6-fold surface area enhancement (186.39 m^2^ g^−1^) together with 3.13 at.% in-situ nitrogen doping, leading to substantially improved interfacial charge transfer and electrocatalytic activity. Through comprehensive structural, physicochemical, and electrochemical characterization, we demonstrate that carbon pore accessibility is a critical structural parameter governing electrolyte transport and active-site utilization in biomass-derived ferrite/carbon composites. Finally, proof-of-concept nitrite sensing is performed to verify that the proposed optimization strategy can be effectively translated into practical electrochemical sensing applications.

## 2. Results and Discussion

### 2.1. Phase Identification and Crystal Structure (XRD)

**Figure 1:**
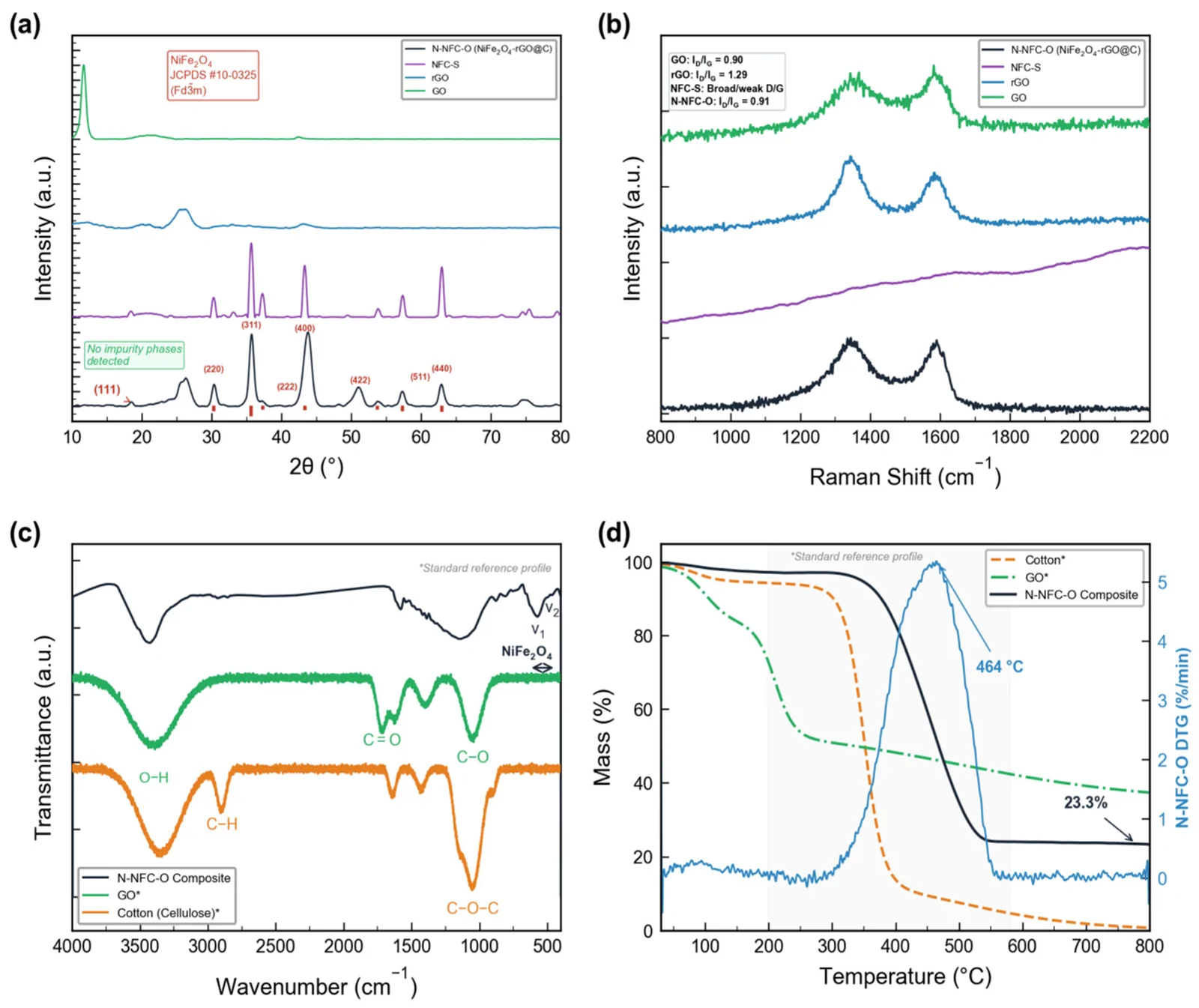
Structural and thermal characterization of NFC-S and N-NFC-O: (**a**) XRD patterns with JCPDS #10-0325 reference for NiFe_2_O_4_ inverse spinel, (**b**) Raman spectra showing D and G bands with I_D/I_G intensity ratios (**c**) FTIR spectra in the 400–4000 cm^−1^ range with reference profiles of cotton cellulose and GO, and (**d**) TGA/DTG curves of N-NFC-O in air (10 °C min^−1^) showing three-stage decomposition with 23.3 wt% NiFe_2_O_4_ residue. The asterisk (*) denotes standard reference profiles. In (**d**), the blue line represents the DTG curve of N-NFC-O (right y-axis, %/min), and the shaded gray region highlights the primary decomposition temperature zone.

Figure 1a shows the XRD patterns of NFC-S and N-NFC-O, with the crystallographic parameters of N-NFC-O summarized in Table 1. The diffraction peaks observed at 2*θ* = 30.30°, 35.72°, 37.10°, 43.82°, 53.50°, 57.20°, and 62.86° can be indexed to the (220), (311), (222), (400), (422), (511), and (440) planes, respectively, of cubic spinel NiFe_2_O_4_ (JCPDS Card No. 10-0325, space group, a = 8.339 Å) [4,30]. The strongest reflection at 35.72°, corresponding to the (311) plane with a d-spacing of 2.51 Å, is characteristic of the spinel ferrite phase.

Application of the Scherrer Equation (1) to the (311) FWHM:

(1) $$D=\frac{K\lambda }{\beta \cos\theta },$$

where *K* = 0.9, *λ* = 1.5406 Å (Cu Kα), *β* is the FWHM in radians, and *θ* is the Bragg angle. The calculated crystallite size of N-NFC-O was approximately 10 nm, markedly smaller than the approximately 24 nm obtained for NFC-S under the same analytical procedure. Despite the higher pyrolysis temperature used for N-NFC-O, the integrated precursor formulation suppressed extensive crystallite coarsening. This behavior may be associated with improved metal-ion dispersion through citrate coordination and with the porous carbon framework generated during urea-assisted pyrolysis, which collectively reduced particle aggregation and sintering [27,31]. Nevertheless, because several synthesis parameters were modified simultaneously, the crystallite-size reduction should be attributed to the integrated optimization strategy rather than to urea alone.

A broad diffraction feature centered at approximately 22–26° is attributed to the overlapping contributions of turbostratically disordered carbon and poorly stacked rGO layers. Its broad profile and low intensity indicate limited long-range ordering of the carbon framework rather than a well-developed graphite structure. No clearly resolved carbon-related (002) feature was observed for NFC-S, possibly because of its highly disordered and carbon-rich matrix. The reference GO sample exhibits a characteristic (001) reflection at approximately 10.6°, corresponding to an interlayer spacing of about 0.83 nm (Figure 1a). The disappearance of this peak after pyrolysis indicates disruption of the regularly stacked oxygenated GO structure and is consistent with substantial thermal reduction of GO.

No diffraction peaks attributable to α-Fe_2_O_3_ (2*θ* = 24.1°, 33.1°), NiO (2*θ* = 37.2°, 43.3°), metallic Fe (2*θ* ≈ 44.7°), Ni (2*θ* ≈ 44.5°), or FeNi alloy (2*θ* ≈ 44.2°, 51.5°) are detected, confirming phase-pure NiFe_2_O_4_ formation. This clean composition validates the corrected Fe:Ni = 2:1 precursor stoichiometry-which ensures quantitative metal incorporation without the amorphous secondary phases that plagued the 1:1 NFC-S formulation-and the 600 °C pyrolysis under N_2_, which provides sufficient thermal energy for complete spinel crystallization while the oxygen-rich micro-environment from cellulose decomposition stabilizes the oxide against carbothermal reduction [26,32]. Pronounced peak broadening (FWHM = 0.8–1.2° after instrumental correction) superimposed on a high amorphous carbon background (~88,000–135,000 counts) is characteristic of nanocrystalline oxide phases dispersed within a predominantly carbonaceous matrix.

### 2.2. Carbon Structure and Defect Analysis (Raman Spectroscopy)

Raman spectroscopy was employed to evaluate the graphitization degree and defect characteristics of the carbon components, as shown in Figure 1b. Two prominent bands appear at ~1343 cm^−1^ and ~1590 cm^−1^. The D band at ~1343 cm^−1^ is associated with the defect-activated breathing mode of six-membered sp^2^ carbon rings, whereas the G band at ~1590 cm^−1^ corresponds to the E_2_g in-plane stretching of sp^2^ C–C bonds [33,34].

The measured (I_D/I_G) ratio for N-NFC-O is 0.91, indicating a defective and partially ordered carbon structure typical of biomass-derived carbons pyrolyzed at 500–700 °C [34]. Compared with NFC-S ((I_D/I_G\approx0.99), with relatively broad and weak D/G features), the slightly lower ratio suggests increased structural ordering of the carbon framework at 600 °C compared with 500 °C. The higher pyrolysis temperature may promote more extensive aromatization and condensation of cellulose-derived carbon, thereby generating larger and more interconnected sp^2^ domains [35]. However, because (I_D/I_G) is also affected by heteroatom incorporation and the structural disorder of carbon, this decrease should be interpreted as a relative structural change rather than definitive evidence of a higher graphitization degree. Concurrently, nitrogen doping derived from urea decomposition may create substitutional and edge defects that contribute to D-band activation, while pyridinic and graphitic nitrogen species may facilitate surface reactivity and electronic transport, respectively [36].

A weak and broad feature near ~2700 cm^−1^ corresponds to the 2D (G′) band, whose low intensity and breadth indicate limited long-range graphitic ordering and a turbostratically disordered carbon structure [36]. For reference, the standalone GO spectrum included in Figure 1b displays an (I_D/I_G) ratio of 0.90, which increases to 1.29 for rGO, consistent with the formation of new and smaller sp^2^ domains during chemical or thermal reduction [37]. The (I_D/I_G) ratio of 0.91 observed for N-NFC-O reflects the combined effects of biomass-derived carbon, rGO, nitrogen-induced defects, and the higher pyrolysis temperature. Of particular importance for electrocatalysis, this defect-containing carbon framework may provide anchoring sites for NiFe_2_O_4_ nanoparticles, promote interfacial electronic coupling, and contribute additional sites for heterogeneous electron transfer reactions [38].

### 2.3. Functional Group Analysis (FTIR Spectroscopy)

Fourier-transform infrared (FTIR) spectroscopy was performed to identify the surface functional groups and provide complementary evidence for the formation of the spinel structure, as presented in Figure 1c.

In the diagnostic low-wavenumber region (400–700 cm^−1^), two characteristic spinel ferrite bands are clearly resolved: ν_1_ ≈ 573 cm^−1^, assigned predominantly to tetrahedral (A)-site metal–oxygen stretching, and ν_2_ ≈ 465 cm^−1^, corresponding to octahedral (B)-site metal–oxygen stretching involving Ni^2+^ and Fe^3+^ [4,39]. The higher wavenumber and stronger intensity of ν_1_ compared with ν_2_ reflect the shorter bond length and larger force constant associated with tetrahedral coordination, which is a characteristic feature of spinel ferrites [39]. These bands provide complementary vibrational evidence for the formation of the NiFe_2_O_4_ spinel structure, consistent with the XRD and SAED results.

At higher wavenumbers, a broad absorption band at ~3430 cm^−1^ is attributed to O–H stretching vibrations of adsorbed water and residual hydroxyl groups on the rGO surface [40]. Bands located at ~1630 cm^−1^ and ~1380 cm^−1^ are assigned to the C=C skeletal vibration of the graphitic carbon framework and residual oxygen-containing functional groups, respectively. Notably, the characteristic C=O stretching band typically observed near ~1720 cm^−1^ for GO is absent after pyrolysis, indicating substantial removal of oxygen-containing functionalities during thermal treatment. Together with the attenuation of the O–H band, these results suggest that the 600 °C pyrolysis effectively promotes the conversion of GO to rGO while retaining a controlled amount of surface oxygen functionalities [41]. The remaining oxygen-containing groups may improve interfacial interaction between the carbon framework and electrolyte without significantly disrupting the electronic conductivity of the rGO network, thereby benefiting subsequent electrochemical reactions [42]. For comparison, representative FTIR reference spectra of pristine cotton cellulose and GO are included in Figure 1c, adapted from characteristic spectral features reported in the literature [24,40].

### 2.4. Morphology and Elemental Distribution (SEM and EDS)

**Figure 2:**
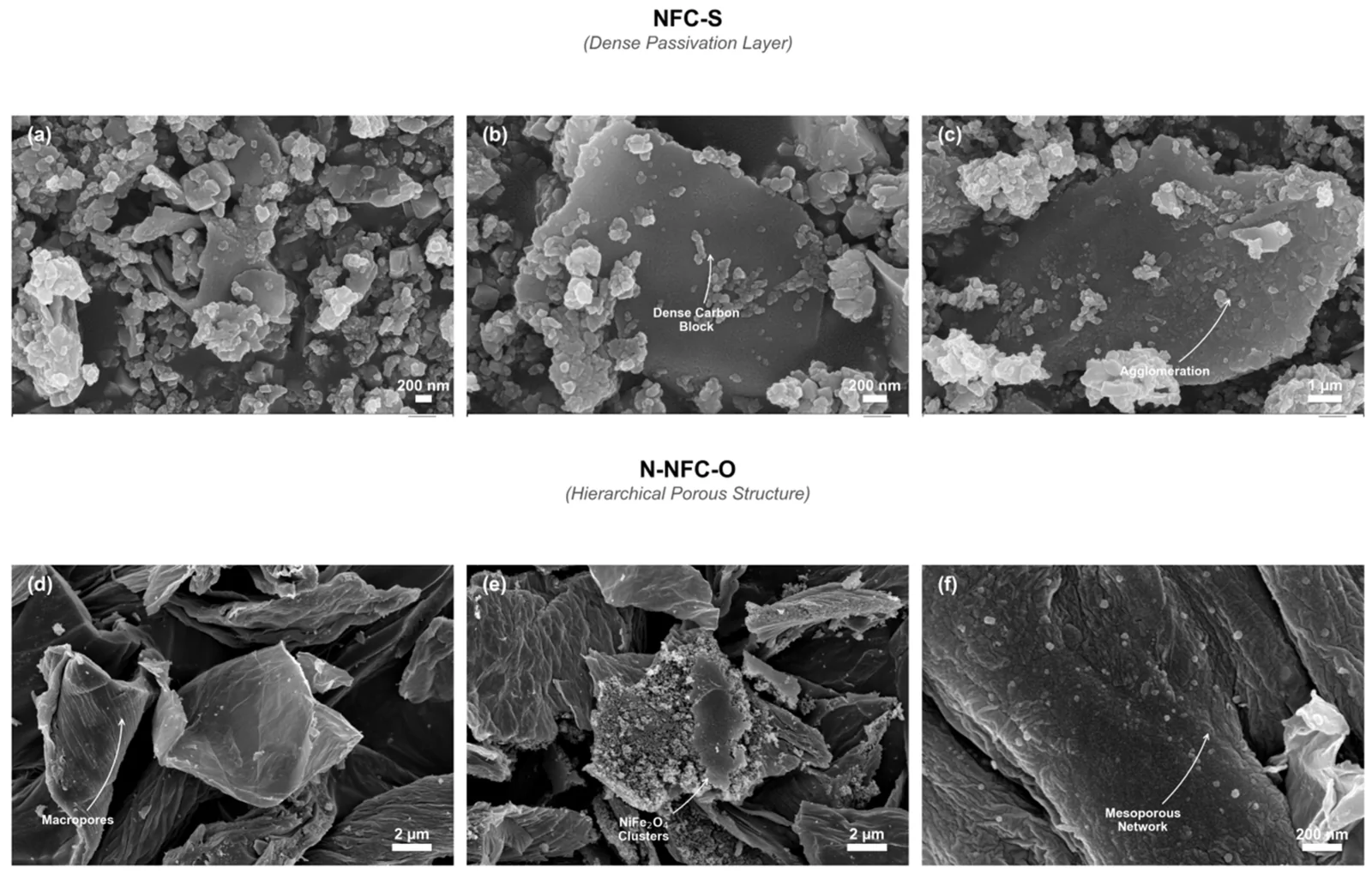
SEM morphology comparison of NFC-S and N-NFC-O composites: (**a**–**c**) SEM micrographs of NFC-S at increasing magnifications showing dense, block-like carbon morphology with extensive agglomeration and embedded NiFe_2_O_4_ nanoparticles; (**d**–**f**) SEM micrographs of N-NFC-O revealing a three-dimensional hierarchical porous architecture with macroporous channels, mesoporous walls, and well-dispersed nanoparticles on carbonized fiber surfaces.

Field-emission scanning electron microscopy reveals distinctly different morphologies for the two composites (Figure 2).

NFC-S (Figure 2a–c) exhibits a dense, block-like carbon morphology with extensive agglomeration and minimal porosity. No discernible fibrous or sheet-like features are present, indicating that the stepwise synthesis and 500 °C pyrolysis caused severe carbon consolidation into a compact monolithic structure. At higher magnifications (Figure 2b,c), the surface is predominantly smooth, with NiFe_2_O_4_ nanoparticles largely buried within the carbon matrix rather than decorating accessible surfaces—a morphology consistent with the carbon-shell passivation phenomenon identified in Section 2.9 [13].

In marked contrast, N-NFC-O (Figure 2d–f) displays a three-dimensional hierarchical porous architecture. An open macroporous network with interconnected channels is visible at low magnification (Figure 2d), while intermediate magnification (Figure 2e) reveals mesoporous walls decorated with NiFe_2_O_4_ nanoparticle clusters and wrinkled rGO nanosheets. High-magnification imaging (Figure 2f) resolves a carbonized fiber framework preserving the original cellulose template morphology, with individual NiFe_2_O_4_ nanoparticles (~20–50 nm) well-dispersed across carbon fibers and rGO surfaces without macroscopic phase segregation [23]. This hierarchical porosity—macropores for electrolyte ingress, mesopores for high surface area, and a conductive carbon scaffold for electron transport—is a direct consequence of urea-derived gas evolution (NH_3_, CO_2_) during pyrolysis [27].

Quantitative EDS analysis of NFC-S yielded Ni = 31.9 wt%, Fe = 29.5 wt%, C = 21.4 wt%, and O = 17.2 wt%, with the Fe:Ni weight ratio of ~0.92:1 reflecting the 1:1 precursor stoichiometry. The high combined metal content (>60 wt%) is consistent with the dense morphology. For N-NFC-O, NiFe_2_O_4_ constitutes only 23.3 wt% (TGA, Section 2.8) dispersed throughout a high-porosity carbon matrix (186.39 m^2^ g^−1^); this low metal loading diluted within a predominantly low-density scaffold yields insufficient signal-to-noise ratios for reliable EDS quantification. Surface composition was characterized by XPS (Section 2.6), whose shallow sampling depth (~5–10 nm) provides more representative analysis of the carbon-rich outer surface [43].

### 2.5. Nanostructure and Crystallography (TEM/HRTEM/SAED)

**Figure 3:**
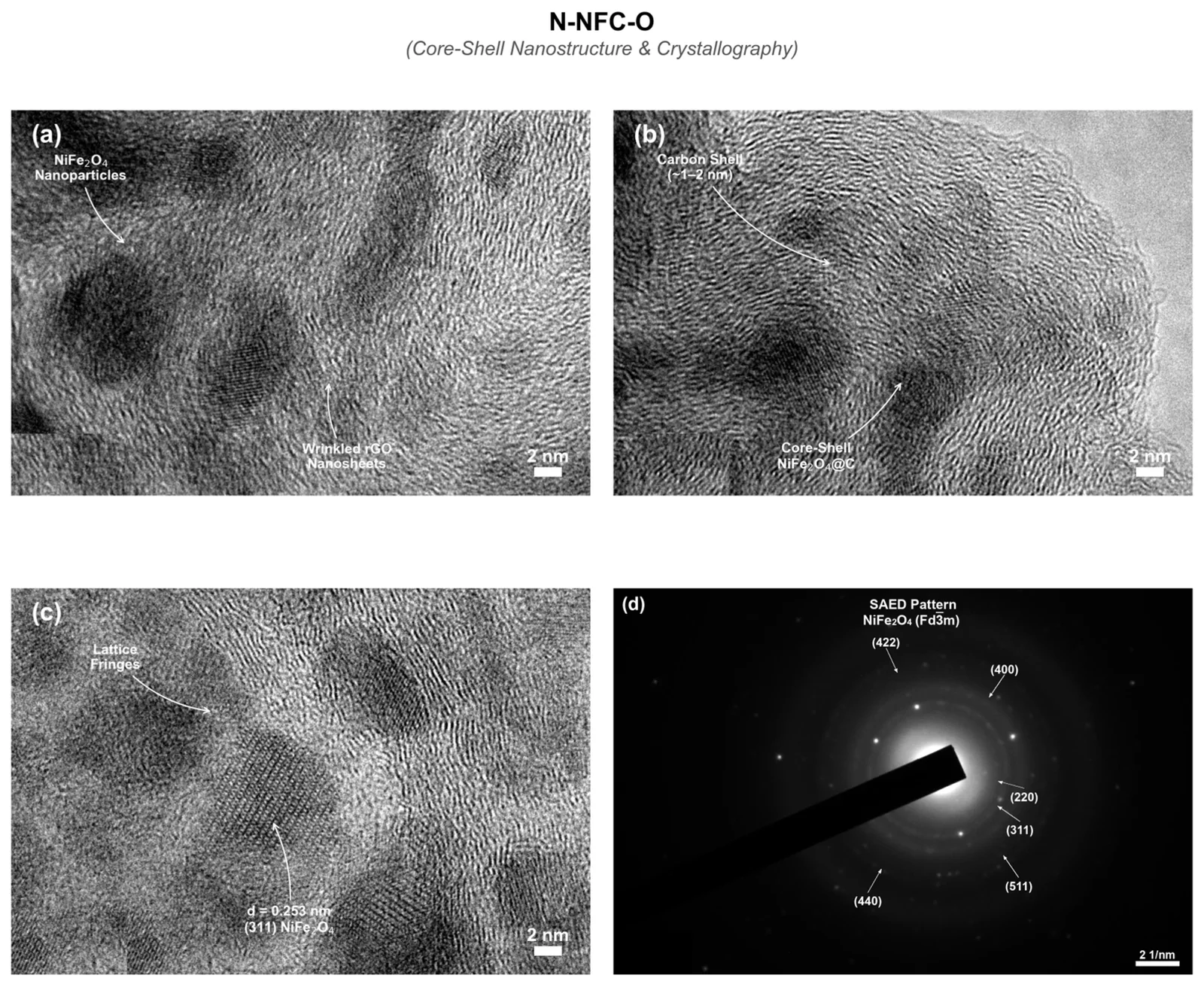
TEM, HRTEM, and SAED characterization of N-NFC-O: (**a**) low-magnification TEM showing wrinkled few-layer rGO sheets decorated with NiFe_2_O_4_ nanoparticle clusters (5–15 nm); (**b**) higher-magnification TEM revealing core–shell NiFe_2_O_4_@C nanostructure with a porous, discontinuous carbon coating (~1–2 nm); (**c**) HRTEM lattice fringes with an interplanar spacing of d = 0.253 nm corresponding to the (311) plane of NiFe_2_O_4_ (JCPDS #10-0325); (**d**) SAED pattern exhibiting polycrystalline diffraction rings indexed to the (220), (311), (400), (422), (511), and (440) planes of the spinel NiFe_2_O_4_ structure.

Transmission electron microscopy was employed to probe the nanoscale architecture of N-NFC-O, as shown in Figure 3a–d.

Low-magnification TEM (Figure 3a) reveals large-area, electron-transparent rGO nanosheets with pronounced wrinkling and folding, suggesting the presence of few-layer rGO sheets consistent with effective exfoliation [41]. Dark-contrast NiFe_2_O_4_ nanoparticle clusters decorate the wrinkled surfaces and fold edges, where high curvature and defect sites serve as preferential nucleation centers. Individual nanoparticles exhibit quasi-spherical morphology with a size distribution of 5–15 nm (predominantly 8–15 nm), uniformly distributed on the carbon support without obvious free-standing particles, suggesting intimate oxide–carbon interfacial contact. The uniform particle distribution is likely associated with citrate-assisted precursor dispersion together with spatial confinement provided by the porous carbon framework, which effectively suppresses excessive particle growth during pyrolysis [44].

A closer examination at higher magnification (Figure 3b) reveals well-defined particle boundaries and several nanoparticles enveloped by a thin, disordered carbon layer (~1–2 nm), forming a core–shell NiFe_2_O_4_@C architecture. Critically, unlike the dense and continuous carbon shells observed in NFC-S, the carbon coating in N-NFC-O appears porous and discontinuous, which is likely related to gas evolution during urea decomposition, creating nanoscale pores within the carbon shell and alleviating passivation while retaining protection against nanoparticle dissolution [34].

HRTEM imaging (Figure 3c) shows continuous, well-ordered lattice fringes extending across the observed nanoparticles, confirming their highly crystalline nature. A measured interplanar spacing of d = 0.253 nm matches the (311) plane of NiFe_2_O_4_ (JCPDS #10-0325, d_311_ = 0.253 nm), providing atomic-scale evidence consistent with the XRD phase identification presented. The corresponding selected-area electron diffraction (SAED) pattern (Figure 3d) exhibits well-defined concentric rings indexing to the (220), (311), (400), (422), (511), and (440) planes of the spinel structure, No additional diffraction rings attributable to detectable crystalline metallic (Fe, Ni, FeNi) or secondary oxide (α-Fe_2_O_3_, NiO) phases are observed, indicating that spinel NiFe_2_O_4_ is the dominant crystalline phase.

The TEM-derived particle size (5–15 nm) is in reasonable agreement with the Scherrer-derived crystallite size (7–10 nm), with the slight discrepancy attributable to TEM measuring the physical particle dimension, including the thin amorphous carbon surface layer, whereas the Scherrer equation probes only the coherent crystalline domain [45]. The ultrafine dimensions provide a high surface-to-volume ratio and abundant exposed active interfaces, which are expected to facilitate electrolyte accessibility and interfacial electron transfer, thereby benefiting subsequent electrochemical reactions [4].

### 2.6. Surface Chemical States and Nitrogen Doping (XPS)

**Figure 4:**
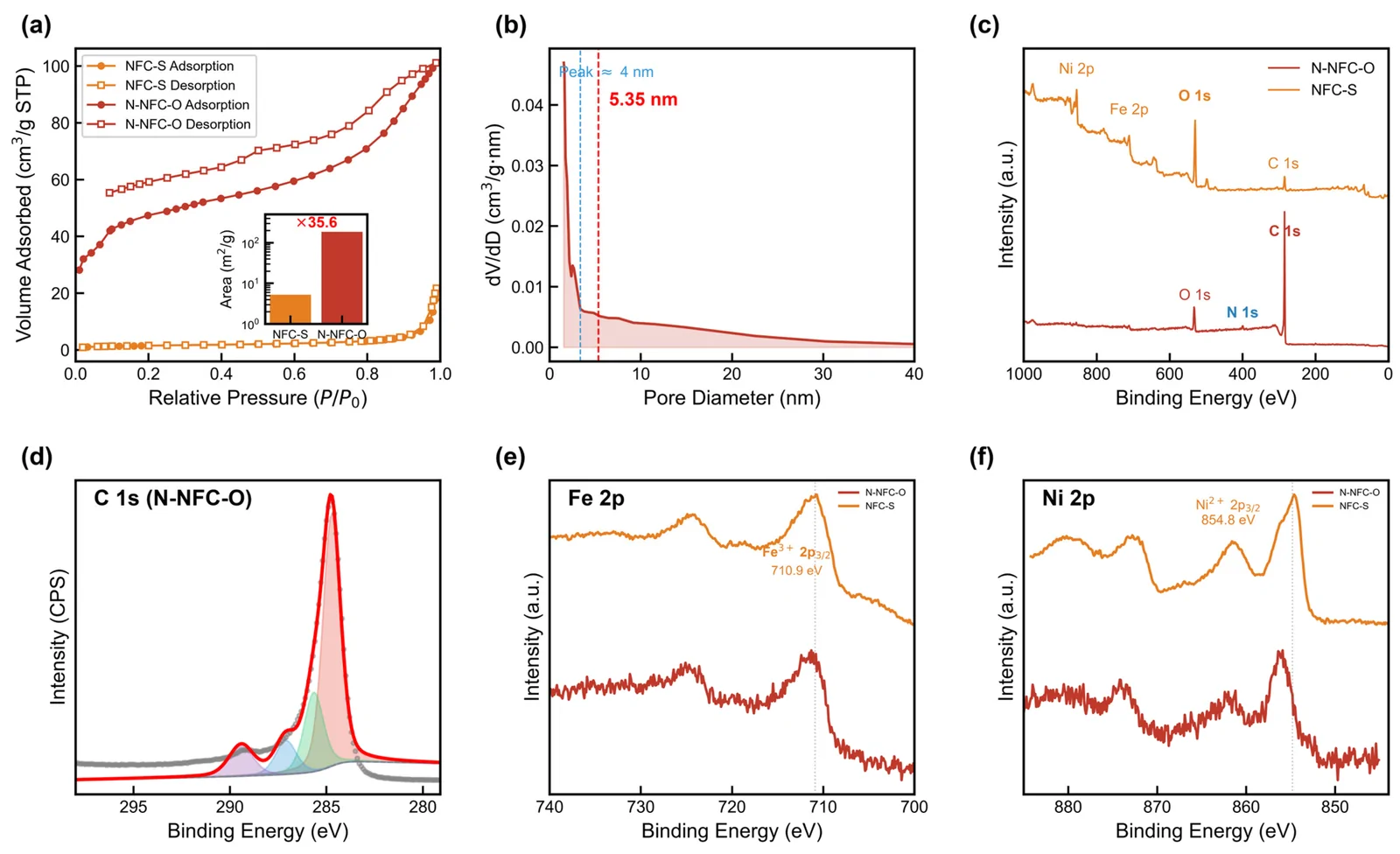
Surface area, pore structure, and surface chemical state analysis: (**a**) N_2_ adsorption–desorption isotherms of NFC-S and N-NFC-O exhibiting Type IV profile with H3-type hysteresis loop (inset: BET surface area comparison, ×35.6 enhancement), (**b**) BJH pore size distribution showing a prominent peak centered at approximately 5.35 nm, (**c**) XPS survey spectra comparing NFC-S and N-NFC-O surface elemental composition, (**d**) high-resolution C 1s spectrum of N-NFC-O with peak deconvolution showing C=C/C–C, C–N, C–O, and O–C=O components, fitted component peaks are shown as colored areas, (**e**) overlaid Fe 2p spectra of NFC-S (Fe^3+^ 2p_3_/_2_ at 710.9 eV) and N-NFC-O (Fe^3+^ 2p_3_/_2_ at 711.9 eV), vertical dashed lines indicate characteristic peak positions, and (**f**) overlaid Ni 2p spectra showing Ni^2+^ 2p_3_/_2_ at 854.8 eV, vertical dashed lines indicate characteristic peak positions.

X-ray photoelectron spectroscopy was performed to determine the surface elemental composition, chemical bonding states, and nitrogen doping configuration. Survey and high-resolution spectra are presented in Figure 4c–f with quantitative elemental analysis summarized in Table 2.

#### 2.6.1. Survey Spectrum and Elemental Composition

Overlaid survey spectra of NFC-S and N-NFC-O (Figure 4c) reveal markedly different surface elemental compositions. For N-NFC-O, five elements are identified: C 1s (284.79 eV, 84.51 at.%), O 1s (532.62 eV, 10.68 at.%), N 1s (400.25 eV, 3.13 at.%), Fe 2p (711.34 eV, 1.08 at.%), and Ni 2p (856.04 eV, 0.60 at.%). NFC-S exhibits a substantially different surface composition, comprising C 1s (30.26 at.%), O 1s (48.85 at.%), Fe 2p (7.99 at.%), and Ni 2p (12.90 at.%), with no detectable N 1s signal.

The combined surface metal content (Fe + Ni) of NFC-S (20.89 at.%) is approximately 12 times higher than that of N-NFC-O (1.68 at.%), whereas its carbon content is considerably lower (30.26 versus 84.51 at.%). Given the surface-sensitive nature of XPS, the high carbon-to-metal ratio of N-NFC-O suggests that the detected surface is predominantly composed of the carbon matrix and that a substantial fraction of the NiFe_2_O_4_ nanoparticles is covered or surrounded by carbon. In contrast, the much stronger Fe and Ni signals of NFC-S indicate a larger proportion of oxide species located within the XPS sampling depth. These results support different near-surface distributions of carbon and NiFe_2_O_4_ in the two composites, although the XPS survey spectra alone cannot determine the continuity, porosity, or exact thickness of the carbon coating. Combined with the TEM observations, the results are consistent with a thin carbon-rich interfacial environment around the nanoparticles in N-NFC-O.

The surface Fe atomic ratio of N-NFC-O is approximately 1.80:1, which is relatively close to the nominal 2:1 Fe ratio of NiFe_2_O_4_. By comparison, NFC-S exhibits a surface Fe ratio of approximately 0.62:1, consistent with its Ni-rich precursor formulation and/or preferential surface enrichment of Ni-containing species. Because XPS probes only the near-surface region and the metal signals of N-NFC-O are relatively weak, these ratios should be interpreted as surface compositional indicators rather than definitive measurements of bulk NiFe_2_O_4_ stoichiometry. The detection of N 1s exclusively in N-NFC-O provides strong evidence that nitrogen was introduced during the urea-assisted synthesis, with urea being the most probable nitrogen source under the present preparation conditions.

#### 2.6.2. C 1s Core-Level Spectrum

Deconvolution of the C 1s spectrum of N-NFC-O (Figure 4d) resolves four components assigned to C=C/C–C at 284.7 eV (59.9%), C–N at 285.7 eV (20.8%), C–O at 287.1 eV (9.6%), and O–C=O at 289.4 eV (9.8%) [46]. The dominant C=C/C–C component indicates that sp^2^-bonded carbon constitutes the major carbon environment, supporting partial restoration and preservation of the conjugated carbon framework during pyrolysis.

#### 2.6.3. O 1s Core-Level Spectrum

The O 1s envelope of N-NFC-O (Figure 4e) is deconvoluted into three contributions: lattice metal–oxygen species at 530.5 eV (13.5%), attributed primarily to the NiFe_2_O_4_ lattice; surface hydroxyl and/or carbonyl-related oxygen at 532.3 eV (49.1%); and C–OH, adsorbed water, and other weakly bound oxygen species at 533.8 eV (37.4%) [46]. Because several oxygen-containing species exhibit overlapping O 1s binding energies, the latter two components are assigned to combined surface oxygen environments rather than to individual functional groups exclusively. The lattice M–O component provides complementary spectroscopic evidence for the presence of metal–oxygen bonding in the composite, consistent with the NiFe_2_O_4_ phase identified by XRD, FTIR, HRTEM, and SAED. The relatively large contribution from surface oxygen species further indicates that oxygen-containing functionalities remain on the carbon framework and oxide surface after pyrolysis, which may facilitate interfacial interaction with the electrolyte and anchoring of the oxide nanoparticles.

#### 2.6.4. Fe 2p Core-Level Spectrum

Overlaid Fe 2p spectra (Figure 4e) show that N-NFC-O exhibits the characteristic spin-orbit doublet with Fe 2p_3_/_2_ at 711.9 eV and a satellite at ~718.5 eV, diagnostic of Fe^3+^ in an oxide environment [47]. NFC-S displays substantially higher Fe 2p intensity (7.99 at.% vs. 1.08 at.%) but essentially identical peak positions—confirming that the NiFe_2_O_4_ phase is chemically equivalent in both samples and that accessibility, not chemistry, differs. The broad peak width (FWHM = 4.95 eV) suggests Fe^2+^/Fe^3+^ coexistence, a hallmark of the inverse spinel structure in which Fe^3+^ ↔ Fe^2+^ electron hopping occurs at the octahedral B-sites [48]. No metallic Fe^0^ component (706.7 eV) is detected, definitively excluding metallic iron phases.

#### 2.6.5. Ni 2p Core-Level Spectrum

Ni 2p spectra (Figure 4f) follow the same trend: NFC-S shows dramatically stronger signals (12.90 at.% vs. 0.60 at.%), with Ni 2p_3_/_2_ at 854.8–856.8 eV indicating Ni^2+^/Ni^3+^ at octahedral B-sites [46]. The binding energy, slightly higher than typical Ni^2+^ in NiO (854.0 eV), suggests mixed Ni^2+^/Ni^3+^ valence arising from the spinel crystal field. No metallic Ni^0^ (852.6 eV) is present. Together with the Fe 2p results, these data establish multiple coexisting redox couples (Fe^2+^/Fe^3+^ and Ni^2+^/Ni^3+^) serving as primary Faradaic electron transfer sites [48].

#### 2.6.6. N 1s Core-Level Spectrum—Key Finding

Detection of nitrogen (3.13 at.%) represents a significant finding. Deconvolution of the N 1s spectrum resolves four configurations: pyridinic-N at 398.6 eV (31.8%), contributing one p-electron to the π-system at edge or defect sites; pyrrolic-N at 400.0 eV (25.8%), incorporated into five-membered rings; graphitic-N (quaternary-N) at 401.0 eV (30.4%), substituting interior carbon atoms; and oxidized-N at 403.2 eV (12.1%) [49].

Nitrogen originates from urea (CO(NH_2_)_2_), initially incorporated solely as a pore-forming agent. During pyrolysis, urea decomposes at ~300 °C to release NH_3_ and HNCO; at 600 °C, these reactive intermediates are captured by the carbon framework through substitutional reactions, achieving in-situ nitrogen doping [27]. This dual functionality—simultaneous pore formation and nitrogen doping—constitutes an unanticipated synergistic benefit of the one-pot design.

Of particular significance, pyridinic-N (31.8%) and graphitic-N (30.4%) together account for over 62% of total nitrogen (3.13 at.%). Pyridinic-N introduces localized electron density near the Fermi level, lowering overpotentials for heterogeneous electron transfer, while graphitic-N enhances electronic conductivity through n-type electron donation [29,50]. Pyrrolic-N (25.8%) contributes pseudocapacitive response through its Lewis basic character. This diverse ensemble of nitrogen-derived active sites complements the Faradaic redox activity of NiFe_2_O_4_, contributing to the observed ΔEp reduction from 113.3 mV (NFC-S, nitrogen-free) to 72.3 mV (N-NFC-O) and the exclusive activation of inner-sphere NO_2_^−^ catalysis in N-NFC-O (Section 2.9) [50].

### 2.7. Pore Structure and Surface Area (BET and BJH Analysis)

N_2_ adsorption–desorption analysis was employed to quantify the specific surface area and N_2_-accessible pore structure relevant to electrolyte–active-site interactions. (Figure 4a) presents the N_2_ adsorption–desorption isotherm of N-NFC-O, which exhibits a characteristic Type IV profile with an H3-type hysteresis loop, according to the IUPAC classification [51]. The hysteresis loop spans (P/P_0 = 0.45–1.0), indicating a well-developed mesoporous architecture with slit-shaped pores commonly associated with aggregates of plate-like carbonaceous particles [52].

The BET-derived specific surface area of N-NFC-O reaches 186.39 m^2^ g^−1^, representing a 35.6-fold enhancement over the stepwise-synthesized NFC-S composite (5.23 m^2^ g^−1^) (Figure 4a, Table 3). This increase constitutes the most significant structural improvement achieved through the multi-parameter optimization strategy. The exceptionally low surface area of NFC-S (5.23 m^2^ g^−1^) was previously attributed to a dense, non-porous carbon shell generated by 500 °C pyrolysis of 1.0 g cotton, which hermetically sealed the embedded NiFe_2_O_4_ nanoparticles and rGO sheets from external access [13]. The dramatic surface area recovery in N-NFC-O can be rationalized by the synergistic effects of three complementary optimization parameters, which collectively promote pore development, carbon-shell regulation, and structural accessibility.

(1) Urea-derived gas-phase pore engineering. Urea undergoes cascade decomposition during thermal processing: melting at ~133 °C, followed by sequential release of NH_3_, HNCO, and CO_2_ at 200–400 °C [27,28]. Gas evolution within the consolidating carbon matrix may act as an in-situ pore-forming process, generating micro- and mesopores and disrupting the dense carbon structure associated with NFC-S passivation.

(2) Reduction of carbon-precursor loading. Decreasing the cotton mass from 1.0 g in NFC-S to 0.40 g in N-NFC-O reduced excessive carbon accumulation that may otherwise restrict pore development. At the higher precursor loading, the larger amount of carbonaceous residue likely promoted the formation of a compact matrix, whereas the five-fold reduction favored the retention of a more open and interconnected carbon architecture.

(3) Elevated pyrolysis temperature. Raising the temperature from 500 °C to 600 °C promotes greater aromatic condensation and structural ordering, as confirmed by the Raman-derived I_D/I_G ratio of 0.91 for partially graphitized carbon with moderate defect density. Higher-temperature carbonization favors a rigid, thermally stable pore skeleton over the amorphous, collapsible matrix produced at 500 °C [35].

Detailed pore structure analysis reveals a hierarchical micro-mesoporous architecture highly favorable for electrochemical applications. The t-plot analysis indicates that micropores (<2 nm) contribute 70.1% of the total surface area, with mesopores accounting for the remainder. The total pore volume was determined to be 0.1564 cm^3^ g−1, with a BET-derived average pore diameter of 3.36 nm and a BJH desorption-derived average pore diameter of 5.35 nm. The BET-derived average pore diameter was calculated using Davg = 4Vtotal/SBET, where Vtotal is the total pore volume and SBET is the BET specific surface area, assuming an equivalent cylindrical pore geometry. Using Vtotal = 0.1564 cm^3^ g^−1^ and SBET = 186.39 m^2^ g^−1^ gives an average pore diameter of approximately 3.36 nm. In contrast, the BJH-derived average pore diameter of 5.35 nm is obtained from the pore-size distribution derived from the desorption branch and is therefore weighted differently toward the mesopore population. A prominent peak centered at approximately 5 nm in the BJH pore size distribution (Figure 4b) confirms the predominance of small mesopores. These pore dimensions substantially exceed the hydrodynamic radii of relevant electrolyte species-hydrated K^+^ (~0.33 nm) and [Fe(CN)_6_]^3−^ (~0.6 nm) [53]-ensuring unimpeded ionic diffusion. The high micropore fraction provides abundant analyte pre-concentration sites, while the mesopore network functions as the primary ion transport highway.

The structure–property relationship is evident: the 35.6-fold increase in BET surface area transforms the carbon matrix from a dense, diffusion-limited framework (NFC-S) into a conductive and hierarchically porous scaffold (N-NFC-O), thereby facilitating electrolyte penetration, increasing active-site accessibility, and accelerating interfacial charge transfer. This remarkable structural evolution arises from the integrated optimization of precursor composition, pore-generation behavior, and carbon-framework reconstruction, which collectively establish a highly accessible conductive architecture.

It should be noted that the optimized N-NFC-O differs from the control NFC-S in multiple synthesis parameters simultaneously, including the Fe:Ni precursor ratio, iron salt identity, cotton loading, chelating agent, pyrolysis temperature, and heating programme, in addition to the introduction of urea. The observed 35.6-fold surface area enhancement therefore reflects an integrated multi-parameter optimization strategy rather than the isolated effect of any single variable. Although the urea-derived gas evolution and nitrogen doping are mechanistically linked to the observed pore formation and N-functionalization (confirmed by XPS and BET), the relative contributions of other modified parameters, particularly the corrected 2:1 Fe:Ni stoichiometry and the elevated pyrolysis temperature, which cannot be independently quantified from the present two-sample comparison.

### 2.8. Thermal Stability and Composition (TGA)

Thermogravimetric analysis (TGA) coupled with derivative thermogravimetry (DTG) was performed in air at a heating rate of 10 °C min^−1^ from room temperature to 800 °C to evaluate the thermal stability and bulk composition of the N-NFC-O NiFe_2_O_4_/rGO@C nanocomposite, as shown in Figure 1d. The TGA profile exhibits a three-stage mass-loss process. In Stage I (room temperature–150 °C), a minor mass loss of 2.94% is attributed mainly to the desorption of physically adsorbed water and residual volatile species from the porous framework. The presence of adsorbed moisture is consistent with the high specific surface area of N-NFC-O (186.39 m^2^ g^−1^), although the amount of water retained also depends on the surface chemistry and storage conditions [51].

The major mass loss occurs during Stage II (200–600 °C), accounting for 72.82% of the initial mass and arising predominantly from oxidative decomposition of the rGO and biomass-derived carbon components. The DTG curve displays a dominant peak centered at approximately 464 °C, which is lower than the typical oxidation temperatures reported for highly graphitized carbon and graphite [34]. This relatively low oxidation temperature is consistent with the defect-containing and partially ordered carbon structure indicated by the Raman (I_D/I_G) ratio of 0.91. The largely overlapping combustion behavior of the carbon components produces a dominant DTG feature, suggesting that the cotton-derived carbon and rGO undergo oxidation over similar temperature ranges; however, complete compositional homogeneity cannot be established from the DTG profile alone.

Above 600 °C, the sample mass gradually stabilizes, leaving a final inorganic residue of 23.30 wt.%, which is attributed predominantly to thermally stable NiFe_2_O_4_ under the applied oxidative conditions [54]. On this basis, the composite contains approximately 23.3 wt.% inorganic oxide and 73.8 wt.% combustible carbonaceous material, with the remaining initial mass loss mainly associated with adsorbed water and volatile species. This bulk mass composition is qualitatively consistent with the carbon-rich surface detected by XPS, although XPS atomic percentages and TGA mass fractions cannot be directly compared because they represent different quantities and sampling depths. The results therefore support a carbon-rich NiFe_2_O_4_ composite architecture in which the carbon framework may provide conductive pathways and help restrict nanoparticle aggregation. For comparison, representative TGA profiles of pristine cotton cellulose and GO are included in Figure 1d, based on characteristic thermogravimetric behavior reported in the literature [24,40].

### 2.9. Electrochemical Performance

Having established the physicochemical characterization of N-NFC-O, a systematic three-electrode comparison was performed using [Fe(CN)_6_]^3−^/^4−^ (5 mM in 1.0 M KCl) as an outer-sphere redox probe for model-independent assessment of interfacial electron transfer kinetics.

#### 2.9.1. Cyclic Voltammetry (CV)

**Figure 5:**
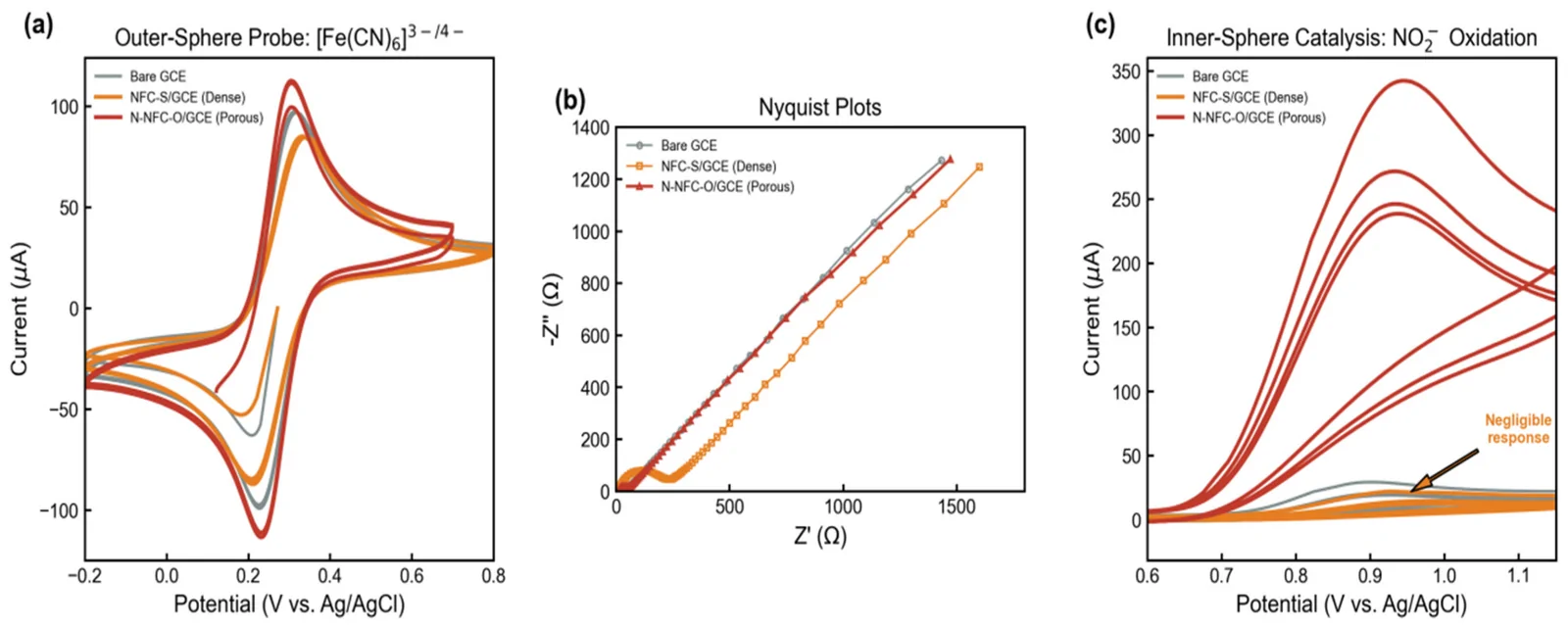
Electrochemical characterization in 5 mM [Fe(CN)_6_]^3−^/^4−^ + 1 M KCl: (**a**) cyclic voltammograms of bare GCE, NFC-S/GCE, and N-NFC-O/GCE at 50 mV s^−1^, (**b**) Nyquist plots from electrochemical impedance spectroscopy (frequency range: 0.1 Hz–100 kHz), and (**c**) differential pulse voltammograms for NO_2_^−^ oxidation in 0.1 M PBS (pH 6.0) showing negligible response for NFC-S and activated catalysis for N-NFC-O.

Figure 5a presents the cyclic voltammograms recorded at 50 mV s^−1^ for three electrode configurations: bare GCE (G1), NFC-S-modified GCE (G2), and N-NFC-O-modified GCE (G3). The bare GCE exhibits the expected quasi-reversible response of the [Fe(CN)_6_]^3−^/^4−^ redox couple, with a peak-to-peak separation (ΔEp) of 71.8 mV, an anodic peak current (Ipa) of 117.89 μA, and an Ipa/Ipc ratio of 1.00, confirming a clean electrode surface and rapid interfacial electron transfer kinetics [55,56,57]. In contrast, NFC-S (G2) displays a markedly deteriorated electrochemical response, with ΔEp increasing to 113.3 mV, Ipa decreasing to 106.35 μA (9.8% reduction), and Ipa/Ipc falling to 0.94, indicating sluggish electron transfer kinetics and reduced electrochemical reversibility [58,59]. Following the one-pot optimization, N-NFC-O (G3) restores the electrochemical behavior to a level nearly identical to that of the bare GCE, exhibiting ΔEp = 72.3 mV, Ipa = 112.60 μA, and Ipa/Ipc = 0.99, demonstrating highly efficient interfacial charge transfer.

The progressive evolution from G2 to G3 establishes a clear structure–property relationship. The severely degraded electrochemical performance of NFC-S is consistent with its extremely low BET surface area (5.23 m^2^ g^−1^), indicating that the dense carbon shell limits electrolyte accessibility and interfacial electron transfer. In contrast, the hierarchically porous N-doped carbon framework of N-NFC-O effectively restores electrochemically accessible interfaces, allowing rapid electrolyte penetration and efficient charge transport throughout the interconnected porous architecture. Consequently, the electrochemical response of G3 approaches that of the bare GCE, demonstrating that improved pore accessibility critically governs charge transfer kinetics in biomass-derived carbon/spinel nanocomposites. In addition to the carbon-shell passivation effect, it should be acknowledged that NFC-S was prepared with a non-stoichiometric 1:1 Fe:Ni precursor ratio, which may independently contribute to its inferior electrochemical response through incomplete spinel phase formation. The observed performance differences between NFC-S and N-NFC-O therefore reflect the cumulative effect of all concurrently modified synthesis parameters rather than any single factor.

The scan-rate dependence further elucidates the charge transfer mechanism. Across the scan-rate range of 10–100 mV s^−1^, the anodic peak current increases linearly with ν^1/2^ (R^2^ > 0.999) for all three electrodes, confirming a diffusion-controlled electrochemical process consistent with the Randles–Ševčík model [60]. The effective electroactive surface area (Aeff) was calculated was calculated using the Randles–Ševčík equation, where n = 1, D = 7.6 × 10^−6^ cm^2^ s^−1^ for [Fe(CN)_6_]^3−^, and C = 5 × 10^−6^ mol cm^−3^. The calculated Aeff values of 0.1326 cm^2^ (G1), 0.1107 cm^2^ (G2), and 0.1301 cm^2^ (G3) reveal a 16.5% reduction in electrochemically accessible surface area following carbon-shell formation and an almost complete recovery (98.1% of the bare GCE) after one-pot optimization. Together with the BET analysis, these results demonstrate that improving pore accessibility reconstructs electrochemically active interfaces, thereby providing the structural basis for the enhanced nitrite oxidation activity discussed in the following section.

#### 2.9.2. Electrochemical Impedance Spectroscopy (EIS)

Electrochemical impedance spectroscopy (EIS) was performed at the formal potential of the [Fe(CN)_6_]^3−^/^4−^ redox couple over the frequency range of 100 kHz–0.01 Hz with a perturbation amplitude of 5 mV to evaluate the interfacial charge transfer resistance (Rct) (Figure 5b). All three electrodes exhibit similar Nyquist profiles consisting of a high-frequency semicircle followed by an inclined Warburg diffusion line. The fitted Rct values are 1419 Ω for bare GCE (G1), 1587 Ω for NFC-S/GCE (G2), and 1459 Ω for N-NFC-O/GCE (G3) (Table 4). Compared with the bare electrode, NFC-S exhibits only a modest 12% increase in Rct, whereas N-NFC-O almost completely restores the interfacial resistance to the original level. Although the differences in Rct are relatively small, they follow the same trend observed in the CV measurements, confirming that the one-pot optimization effectively improves interfacial electron transfer kinetics.

Interestingly, the electrochemical differentiation revealed by CV is substantially more pronounced than that observed by EIS. While the Rct of NFC-S increases by only 12%, its peak-to-peak separation (ΔEp) increases by 57% relative to the bare GCE. This difference reflects the distinct information provided by the two techniques. The outer-sphere [Fe(CN)_6_]^3−^/^4−^ redox probe is primarily sensitive to the macroscopic electrode/electrolyte interface, whereas ΔEp is strongly influenced by electrolyte accessibility and the effective electron transfer kinetics within the porous modifier layer [59]. The significantly larger increase in ΔEp therefore indicates that passivation mainly limits electrolyte transport and active-site accessibility rather than introducing severe bulk electronic resistance.

This interpretation is further supported by the nitrite electrocatalysis results (Figure 5c). Compared with the bare GCE, N-NFC-O/GCE exhibits an approximately 70 mV negative shift in the nitrite oxidation potential, whereas NFC-S/GCE shows negligible electrocatalytic activity under identical conditions. Together with the BET, CV, and EIS analyses, these results demonstrate that the hierarchically porous N-doped carbon framework effectively reconstructs electrochemically accessible interfaces, enabling efficient electrolyte penetration and rapid charge transfer to the embedded NiFe_2_O_4_ active sites. Consequently, carbon pore accessibility emerges as the decisive factor governing the electrocatalytic performance of the biomass-derived ferrite/carbon nanocomposite.

#### 2.9.3. Long-Term Cycling Stability

Practical reliability of the modifier layer was assessed by continuous CV cycling over 1000 cycles at 100 mV s^−1^ in 5 mM [Fe(CN)_6_]^3−^/^4−^ + 1.0 M KCl. The N-NFC-O/GCE electrode retains > 95% of its initial anodic peak current with stable ΔEp throughout (Figure 5a, inset), confirming that the porous N-doped carbon framework maintains structural integrity without delamination or degradation of the NiFe_2_O_4_ active phase. This durability is attributed to strong interfacial bonding from in-situ one-pot synthesis and the mechanical robustness of the partially graphitized carbon skeleton [59]. It should be noted that this stability assessment employed the outer-sphere [Fe(CN)_6_]^3−^/^4−^ probe; long-term stability under repeated NO_2_^−^ analyte exposure was not separately evaluated and remains to be characterized in future sensor-focused studies.

### 2.10. Preliminary Sensor Validation: DPV Detection of Nitrite

**Figure 6:**
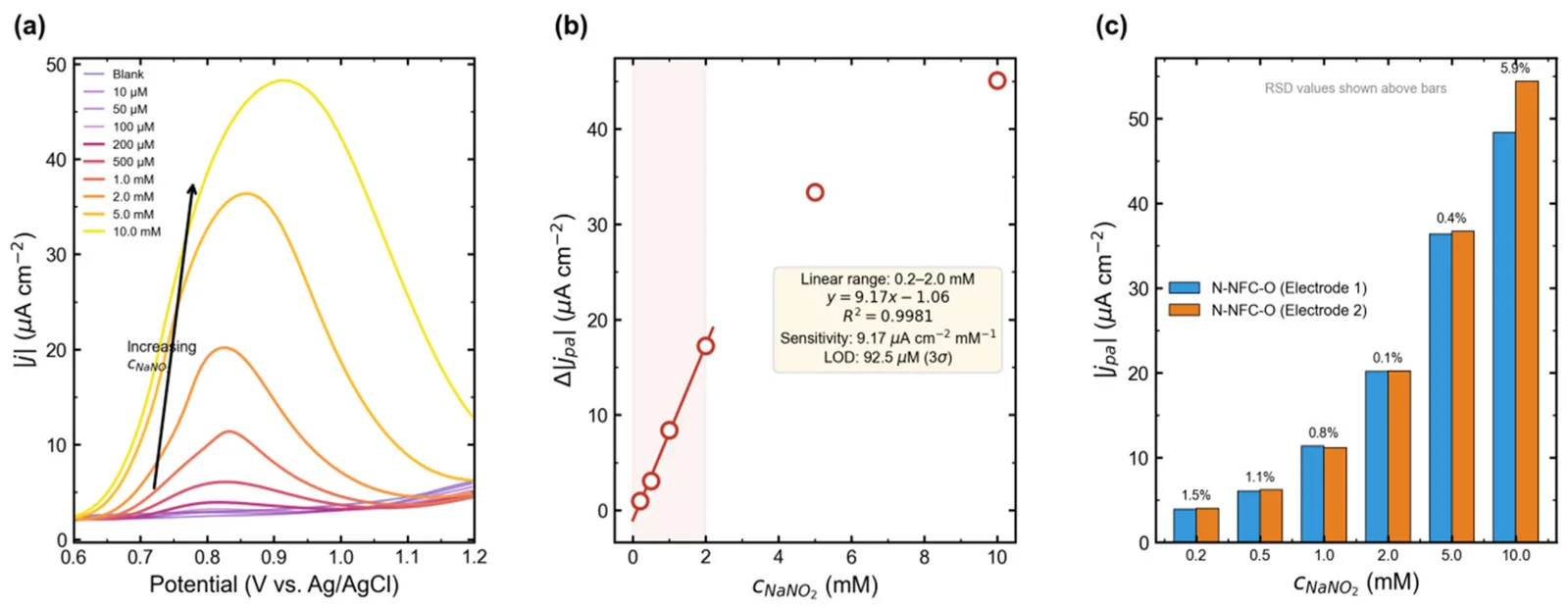
Nitrite sensing performance of N-NFC-O/GCE in 0.1 M PBS (pH 6.0): (**a**) DPV responses at NaNO_2_ concentrations from 10 µM to 10.0 mM, (**b**) calibration curve showing a linear range of 0.2–2.0 mM (sensitivity: 9.17 µA cm^−2^ mM^−1^, R^2^ = 0.9981, LOD: 92.5 µM at 3σ), the shaded pink area indicates the linear detection range (0.2–2.0 mM), and (**c**) electrode-to-electrode reproducibility comparison between two independently fabricated N-NFC-O/GCE electrodes with RSD values labeled for each concentration.

To validate the electroanalytical capability of the optimized nanocomposite, differential pulse voltammetry (DPV) was employed for quantitative detection of nitrite (NO_2_^−^) in 0.1 M phosphate buffer solution (pH 6.0). As shown in Figure 6a, the N-NFC-O/GCE electrode exhibits a well-defined oxidation peak at approximately +0.85 V (vs. Ag/AgCl), and the peak current increases progressively with NaNO_2_ concentration from 10 μM to 10.0 mM, confirming efficient electrocatalytic oxidation of NO_2_^−^ to NO_3_^−^. The enhanced catalytic response originates from the synergistic coupling between the electrochemically accessible NiFe_2_O_4_ active sites and the hierarchically porous N-doped carbon framework, which facilitates electrolyte diffusion, active-site exposure, and rapid interfacial electron transfer. As summarized in Figure 6b, the oxidation current density exhibits an excellent linear relationship with nitrite concentration over the range of 0.2–2.0 mM:

Δ|j_pa_|(µA cm^−2^) = 9.17 × C_NaNO_2_(mM) − 1.06, R^2^ = 0.9981, (2)

corresponding to a sensitivity of 9.17 μA cm^−2^ mM^−1^ and a limit of detection (LOD) of 92.5 μM (3σ/S criterion).

The reproducibility of the optimized electrode was evaluated using two independently prepared N-NFC-O/GCE electrodes over the entire concentration range (Figure 6c). Within the linear detection range (0.2–2.0 mM), the average relative standard deviation (RSD) between the two electrodes is only 0.9%, demonstrating the excellent reproducibility of the one-pot synthesis strategy and the high uniformity of the resulting nanocomposite coating. The stable electrochemical response further confirms that the optimized porous architecture can be reproducibly fabricated without sacrificing electrode-to-electrode consistency. While the highly consistent response (RSD ≈ 0.9%) between these two independently prepared electrodes indicates excellent preliminary reproducibility, it is important to note that a comprehensive evaluation involving multiple independently synthesized material batches remains necessary to fully establish batch-to-batch consistency. Such systematic large-scale statistical validation is identified as a critical direction for future analytical development. The DPV voltammograms recorded specifically within the linear detection range (0.2–2.0 mM) are presented in Figure S1 (Supplementary Materials).

Together with the BET, cyclic voltammetry, and electrochemical impedance analyses, the DPV results provide direct functional validation of the proposed materials design strategy. The pronounced electrocatalytic activity observed for N-NFC-O, in sharp contrast to the negligible response of NFC-S under identical conditions, demonstrates that improving pore architecture effectively translates the structural advantages of the optimized nanocomposite into measurable electrochemical performance gains, reinforcing the central role of electrolyte-accessible pathways in governing interfacial charge transfer and electrocatalytic activity.

We emphasize that the present DPV results constitute preliminary proof-of-concept evidence demonstrating the electroanalytical potential of the N-NFC-O composite. A comprehensive analytical validation including interference testing with common coexisting species (Cl^−^, SO_4_^2−^, NO_3_^−^, K^+^, Na^+^, ascorbic acid, uric acid, dopamine), real-sample analysis in environmental or food matrices, standard addition recovery studies, and long-term storage and operational stability assessments which remains necessary before practical sensor deployment can be considered. These validation studies are beyond the primary scope of the present work, which focuses on identifying and overcoming the passivation phenomenon, and will be addressed in future investigations. Accordingly, the present dataset does not include a systematic interference study under realistic sample conditions. A rigorous evaluation of potential interfering species in representative real-sample matrices will be required in future work before the practical analytical application of this sensor can be established. Accordingly, the present dataset does not include a systematic interference study under realistic sample conditions. A rigorous evaluation of potential interfering species in representative real-sample matrices will be required in future work before the practical analytical application of this sensor can be established.

### 2.11. Structure–Property–Performance Correlation

The structural, physicochemical, and electrochemical characterizations consistently establish a clear structure–property–performance relationship, demonstrating that carbon pore accessibility is the key structural descriptor governing the electrocatalytic behavior of biomass-derived carbon/spinel nanocomposites. The contrasting electrochemical responses of NFC-S and N-NFC-O represent two distinct evolutionary pathways. In NFC-S, the dense carbon shell (BET = 5.23 m^2^ g^−1^) restricts electrolyte penetration, resulting in sluggish ion transport, enlarged peak separation (ΔEp = 113.3 mV), increased charge transfer resistance (Rct = 1587 Ω), and ultimately negligible inner-sphere electrocatalytic activity. In contrast, the hierarchically porous N-doped carbon framework of N-NFC-O (BET = 186.39 m^2^ g^−1^) establishes continuous ion- and electron-transport pathways, restoring rapid interfacial charge transfer (ΔEp = 72.3 mV; Rct = 1459 Ω) and lowering the nitrite oxidation overpotential by approximately 70 mV. Together, these results demonstrate that transforming the carbon matrix from a diffusion-limited shell into an electrolyte-accessible conductive framework fundamentally determines electrocatalytic activation.

The remarkable activation of N-NFC-O originates from the synergistic optimization of multiple structural features rather than any individual parameter. The hierarchically interconnected micro/mesoporous architecture (BET/BJH) dramatically increases electrolyte-accessible surface area, while 3.13 at.% in-situ nitrogen doping (XPS) tailors the electronic structure of the carbon framework and strengthens electronic coupling with adjacent NiFe_2_O_4_ nanoparticles [30]. Simultaneously, refined crystallite dimensions (7–10 nm, XRD/TEM) expose a greater density of catalytically active sites, the near-stoichiometric Fe:Ni surface ratio (1.80:1, XPS) minimizes electrochemically inactive nickel-rich domains, and the partially graphitized carbon framework (ID/IG = 0.91, Raman; DTG peak = 464 °C, TGA) achieves an effective balance between electrical conductivity and structural defects for nanoparticle anchoring. Rather than acting independently, these structural characteristics collectively reconstruct a highly accessible conductive framework that promotes efficient electrolyte diffusion, rapid charge transfer, and effective utilization of embedded NiFe_2_O_4_ active sites.

**Figure 7:**
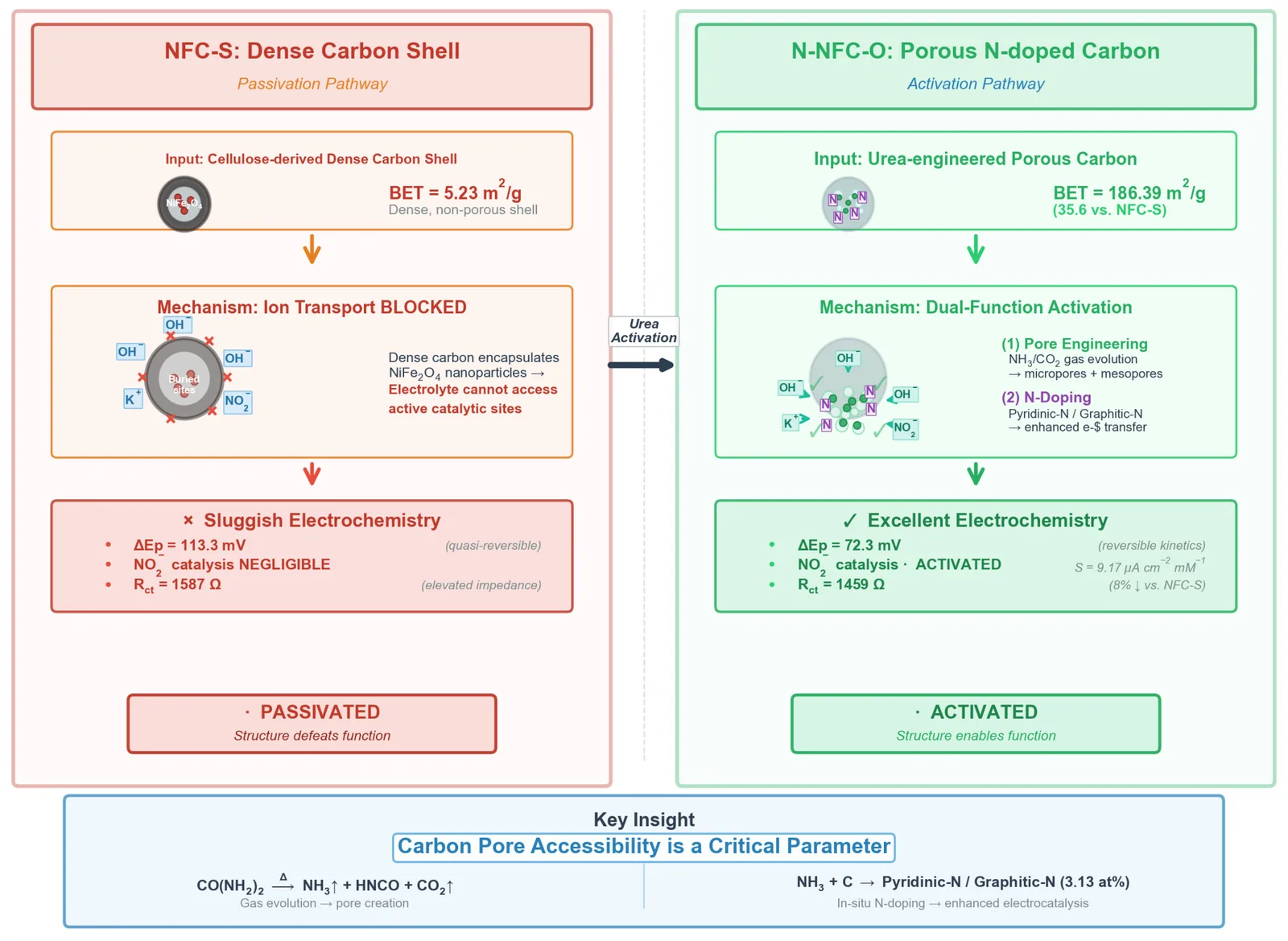
Structure–property–performance correlation diagram comparing NFC-S (carbon-shell passivated) and N-NFC-O (pore-activated). Multi-technique evidence from XRD, Raman, XPS, BET, and electrochemistry converges to identify carbon pore accessibility as a critical parameter governing electrocatalytic activity. Red and green background panels represent the passivation and activation pathways, respectively. The ✗ and ✓ symbols indicate blocked and functional electrochemistry. Downward arrows show the diagnostic progression from structural input to electrochemical outcome. The horizontal arrow labeled ‘Urea Activation’ indicates the optimization strategy connecting the two pathways.

Importantly, the proposed mechanism is consistently supported by complementary characterization techniques. The optimized carbon framework is independently verified by Raman spectroscopy, TGA, and XPS, while the phase purity and crystallographic integrity of NiFe_2_O_4_ are confirmed by XRD, FTIR, XPS, HRTEM, and SAED. The excellent agreement among these independent measurements provides robust cross-validation of the proposed activation mechanism. Accordingly, Figure 7 illustrates the overall structure–property–performance relationship established in this work. In the NFC-S pathway, the dense carbon shell encapsulates NiFe_2_O_4_ nanoparticles and blocks electrolyte access, resulting in severe interfacial passivation [32]. In contrast, the urea-engineered hierarchical pore network in N-NFC-O creates continuous ion-diffusion channels, while the N-doped conductive framework enables rapid electron transport to the embedded active sites, thereby converting the passivated architecture into a highly efficient electrocatalytic platform.

Furthermore, the analytical performance of the proposed N-NFC-O sensor is compared with previously reported non-precious metal or carbon-based nitrite sensors in Table 5. While the absolute limit of detection is higher than those employing noble metals (e.g., AuNPs) or highly conductive 2D materials (e.g., MXene), our sensor provides a highly competitive wide linear range entirely based on low-cost biomass templates and earth-abundant transition metals.

## 3. Materials and Methods

### 3.1. Materials and Reagents

Graphene oxide (GO) powder (single-layer content >90%, lateral dimension 0.5–8 µm, thickness ~0.8 nm) was purchased from Suzhou Tanfeng Graphene Technology Co., Ltd. (Suzhou, China). Iron(III) chloride hexahydrate (FeCl_3_·6H_2_O, ≥99.0%), Iron(III) nitrate nonahydrate (Fe(NO_3_)_3_·9H_2_O, ≥98.5%), nickel(II) nitrate hexahydrate (Ni(NO_3_)_2_·6H_2_O, ≥98.0%), L-ascorbic acid (C_6_H_8_O_6_, ≥99.7%), citric acid monohydrate (C_6_H_8_O_7_·H_2_O, ≥99.5%), urea (CO(NH_2_)_2_, ≥99.0%), and sodium hydroxide (NaOH, ≥96.0%) were obtained from Sinopharm Chemical Reagent Co., Ltd. (Shanghai, China) (all AR grade) and used as received. Medical-grade defatted absorbent cotton (cellulose content >95%) was sourced from local pharmaceutical suppliers. High-purity nitrogen (N_2_, ≥99.99%), absolute ethanol (≥99.7%), Nafion perfluorinated resin solution (5 wt%), and deionized water (≥18.2 MΩ·cm) were used throughout.

### 3.2. Synthesis of NiFe_2_O_4_/rGO@C via Stepwise Method (NFC-S, Control)

**Figure 8:**
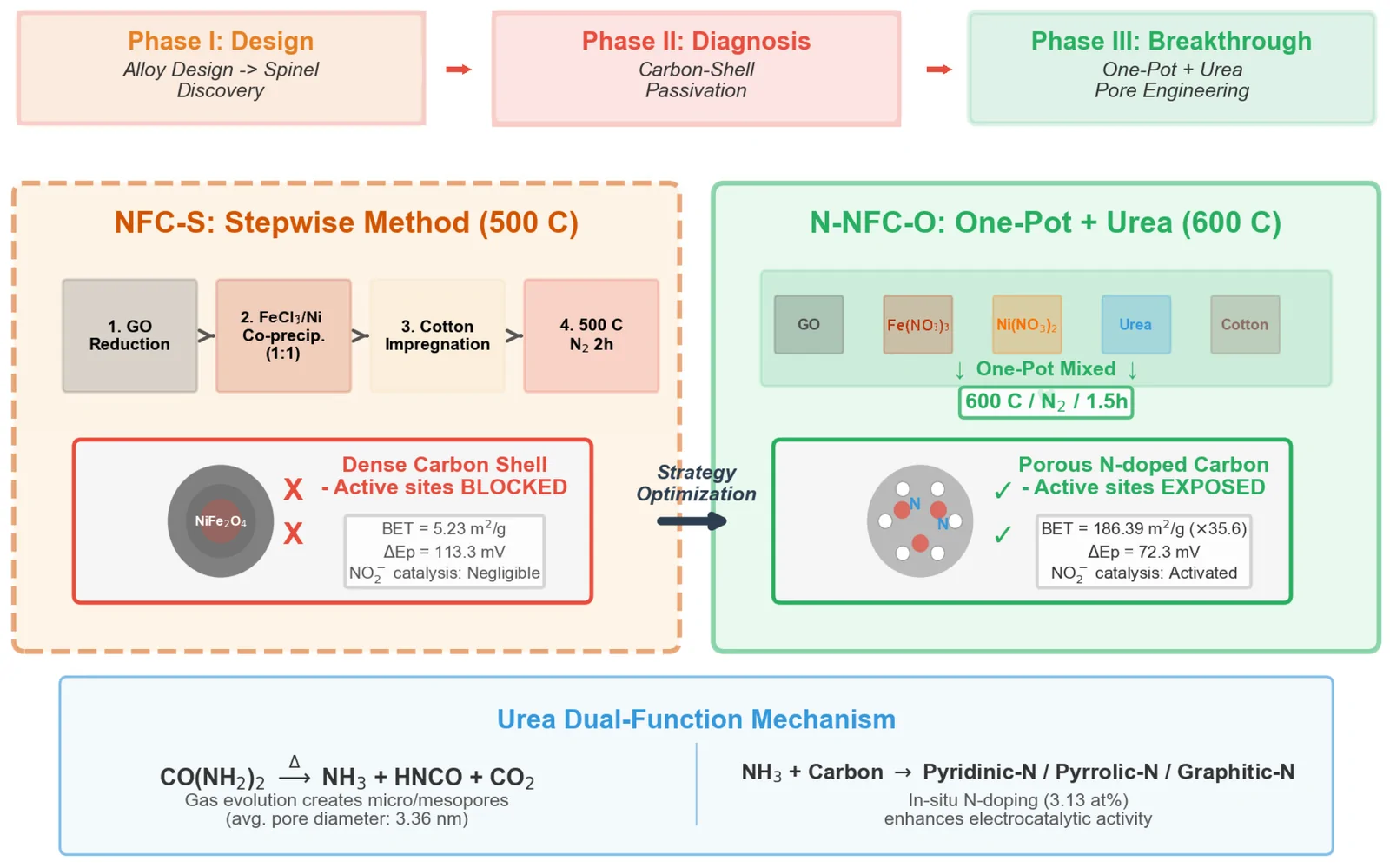
Schematic illustration of the synthesis evolution: (**left**) NFC-S prepared by a five-step stepwise method at 500 °C with Fe:Ni = 1:1, and (**right**) N-NFC-O prepared by a urea-assisted one-pot method at 600 °C with Fe:Ni = 2:1. The (**bottom**) panel shows the urea dual-function mechanism: gas evolution creates micro/mesopores while NH_3_ enables in-situ nitrogen doping. The top panel illustrates the three-phase research strategy. Red ✗ and green ✓ symbols indicate blocked and exposed active sites, respectively. The horizontal arrow represents the strategy optimization from stepwise (NFC-S) to one-pot (N-NFC-O) synthesis.

The stepwise-synthesized composite (NFC-S) was prepared through a conventional biomass-templated route, as illustrated in Figure 8. The synthesis was originally designed using an equimolar Fe:Ni precursor composition together with GO, and cotton as the biomass template. The procedure is summarized below.

GO powder (50 mg) was dispersed in 100 mL of DI water by ultrasonication (1 h). L-Ascorbic acid (200 mg) was added and the mixture was heated at 90 °C for 3 h under magnetic stirring until the suspension turned black. The product was collected by centrifugation (4000 rpm, 20 min), washed three times with ethanol/water, and dried overnight. FeCl_3_·6H_2_O (135.2 mg, 0.5 mmol) and Ni(NO_3_)_2_·6H_2_O (145.4 mg, 0.5 mmol) were dissolved in 20 mL of DI water with ~50 mg L-ascorbic acid. The dried rGO was re-dispersed in 50 mL of DI water (ultrasonication, 30 min). The metal precursor solution was added dropwise under vigorous stirring (30 min), and the pH was adjusted to ~9.5 with 0.1 M NaOH. Defatted absorbent cotton (1.0 g), cut into 0.5 cm^3^ segments, was immersed in the composite slurry, ultrasonicated for 20 min and stirred at room temperature for 1 h. The impregnated cotton was removed, gently blotted, and dried at 60 °C in a forced-air oven for 12 h. The dried precursor was pyrolyzed in a horizontal tube furnace under flowing N_2_ (200 sccm). The temperature was ramped directly from room temperature to 500 °C at 5 °C/min and held for 2 h. The resulting black monolithic composite was collected after cooling to room temperature under N_2_.

### 3.3. Synthesis of N-Doped NiFe_2_O_4_/rGO@C via One-Pot Optimized Method (N-NFC-O)

Compared with NFC-S, the optimized synthesis employed a stoichiometric Fe:Ni precursor ratio (2:1), introduced urea as the nitrogen source and pore-forming agent, reduced the cotton template mass (from 1.0 to 0.40 g). The overall synthesis procedure is illustrated in Figure 8, with a complete parameter comparison in Table 6. These specific synthesis parameters (including the elevated pyrolysis temperature and the cotton mass) were selected based on preliminary exploratory experiments designed to balance oxide crystallinity with carbonization degree, guided by relevant literature on urea-assisted pyrolysis.

All reagents-GO powder (50 mg), Fe(NO_3_)_3_·9H_2_O (404.0 mg), Ni(NO_3_)_2_·6H_2_O (145.4 mg), citric acid monohydrate (315 mg), and urea (250 mg)-were combined in 30 mL of DI water and stirred magnetically for 30 min. Defatted absorbent cotton (0.40 g), cut into ~0.5 cm^3^ segments, was immersed in the precursor solution for 6 h with periodic manual stirring at 0.5, 1.0, 2.0, and 4.0 h intervals. The impregnated cotton was dried at 80 °C for 12 h in a vacuum oven.

The dried precursor was pyrolyzed under flowing N_2_ (200 sccm) with a three-stage program: (i) RT → 120 °C at 2 °C min^−1^, held 30 min; (ii) 120 → 300 °C at 2 °C/min, held 45 min (urea decomposition window—concurrent nitrogen doping and in situ pore formation); (iii) 300 → 600 °C at 5 °C min^−1^, held 1.5 h (cellulose carbonization, GO thermal reduction to rGO, and NiFe_2_O_4_ spinel crystallization). After cooling under N_2_, the product was ground in an agate mortar, washed three times with DI water by centrifugation (6000 rpm, 10 min per cycle), rinsed once with absolute ethanol, and vacuum-dried at 60 °C for 12 h. The resulting black powder was denoted as N-NFC-O.

### 3.4. Material Characterization

Phase composition and crystal structure were characterized by X-ray diffraction (XRD, Bruker D8 Advance, Bruker AXS GmbH, Karlsruhe, Germany, Cu *K*α, *λ* = 0.15406 nm) over 2*θ* = 10–80° (step size 0.02°, scan rate 2° min^−1^). Crystallite sizes were estimated from the Scherrer equation applied to the (311) reflection. Raman spectra were collected on a Renishaw inVia Raman microscope (Renishaw plc, Wotton-under-Edge, UK) equipped with a 532 nm laser. Fourier-transform infrared (FTIR) spectra were recorded on a Thermo Scientific Nicolet iS10 spectrometer (Thermo Fisher Scientific, Madison, WI, USA; 400–4000 cm^−1^, KBr pellet method).

Surface morphology was examined by field-emission scanning electron microscopy (FE-SEM, Carl Zeiss Microscopy GmbH, Jena, Germany. Zeiss SIGMA HD, 5 kV) equipped with energy-dispersive X-ray spectroscopy (EDS). Microstructure and lattice fringes were investigated by transmission electron microscopy (TEM, FEI Talos F200X G2, Thermo Fisher Scientific (FEI), Hillsboro, OR, USA, 200 kV) supplemented by selected-area electron diffraction (SAED).

Surface chemical composition and electronic states were characterized by X-ray photoelectron spectroscopy (XPS, Thermo Fisher Scientific ESCALAB 250Xi, East Grinstead, UK; monochromatic Al Kα, 400 µm spot). All binding energies were calibrated to the adventitious C 1s peak at 284.8 eV; high-resolution spectra were fitted using Gaussian–Lorentzian mixed functions after Shirley background subtraction.

Specific surface area and pore characteristics were determined from N_2_ adsorption–desorption isotherms at 77 K (Micromeritics Instrument Corp., Norcross, GA, USA. Micromeritics TriStar II Plus 3030) after degassing at 200 °C under vacuum for 4 h. The BET method was applied over P/P_0_ = 0.05–0.30; pore size distributions were derived via the BJH model, and micropore volumes were evaluated by the t-plot method.

Thermal stability and composition were evaluated by thermogravimetric analysis coupled with differential scanning calorimetry (TGA-DSC, NETZSCH STA 449F3, NETZSCH-Gerätebau GmbH, Selb, Germany) in air at 10 °C min^−1^ from 30 to 800 °C, with α-Al_2_O_3_ as the reference.

### 3.5. Electrochemical Measurements

Electrochemical measurements were performed on a Corrtest CS100ME electrochemical workstation (Wuhan Corrtest Instruments Corp., Ltd., Wuhan, China) in a three-electrode configuration: a bare or modified glassy carbon electrode (GCE, Ø 3 mm) as the working electrode, a platinum wire counter electrode, and an Ag/AgCl (saturated KCl) reference electrode. All potentials are referenced to Ag/AgCl unless otherwise stated.

The bare GCE was sequentially polished with 1.0, 0.3, and 0.05 µm alumina slurries, ultrasonicated in DI water and ethanol (3 min each), and dried under N_2_. Catalyst ink was prepared by dispersing the nanocomposite powder (1 mg mL^−1^) in absolute ethanol containing 0.5 wt% Nafion via ultrasonication (30 min). A 5 µL aliquot was drop-cast onto the polished GCE and dried at room temperature for 2 h, yielding a catalyst loading of ~0.071 mg cm^−2^. Three electrode configurations were evaluated: G1 (bare GCE); G2 (NFC-S/GCE); G3 (N-NFC-O/GCE).

Cyclic voltammetry (CV) was conducted in 5 mM K_3_[Fe(CN)_6_]/K_4_[Fe(CN)_6_] (1:1) with 1 M KCl supporting electrolyte, scanning from −0.2 to 0.6 V at rates of 10–200 mV s^−1^. The effective electroactive area (A_eff) was estimated from the Randles–Ševčík equation using the known diffusion coefficient of [Fe(CN)_6_]^3−^ in 1 M KCl (D = 7.6 × 10^−6^ cm^2^ s^−1^).

Electrochemical impedance spectroscopy (EIS) was performed in the same electrolyte at open circuit potential over 100 kHz–0.01 Hz with a 5 mV AC perturbation. Impedance data were fitted to a modified Randles circuit. Long-term cycling stability was assessed through 1000 consecutive CV cycles at 100 mV s^−1^.

Differential pulse voltammetry (DPV) was conducted in 0.1 M K-PBS (pH 6.0) with a potential window of 0.6–1.2 V vs. Ag/AgCl, step potential of 4 mV, pulse amplitude of 50 mV, pulse width of 0.05 s, sample width of 0.0167 s, and quiet time of 2 s. NaNO_2_ was sequentially added using the standard addition method over concentrations ranging from 10 µM to 10.0 mM. The optimal pH of 6.0 was determined from a prior pH screening study (pH 4.0–8.0), in which peak current density reached a maximum at pH 6.0 for two independently prepared electrodes.

## 4. Conclusions

A rational multi-parameter optimization strategy was developed to overcome carbon-shell passivation in biomass-templated NiFe_2_O_4_/rGO@C nanocomposites. Compared with the stepwise-synthesized NFC-S, the optimized N-NFC-O composite exhibited a 35.6-fold increase in BET surface area, a hierarchically porous N-doped carbon framework, and substantially improved electrochemical performance. The optimized architecture effectively restored interfacial charge transfer kinetics, recovered electrochemically accessible surface area, and enabled efficient nitrite electrocatalysis, demonstrating that the structural optimization successfully translated into functional electrochemical performance.

More importantly, comprehensive structural and electrochemical characterization consistently established carbon pore accessibility as the key structural descriptor governing the electrocatalytic behavior of biomass-derived ferrite/carbon nanocomposites. Rather than being determined by crystallinity or composition alone, electrochemical activation arises from the synergistic optimization of pore architecture, carbon electronic structure, electrolyte accessibility, and interfacial charge transfer. These findings provide direct experimental evidence that engineering the carbon framework is an effective strategy for eliminating passivation while maximizing utilization of embedded oxide active sites.

Beyond the present NiFe_2_O_4_/rGO@C system, the proposed structure–property–performance relationship provides a general design principle for biomass-derived carbon/oxide hybrid materials. The multi-parameter optimization strategy presented here, for which the nitrite sensing results serve as preliminary proof-of-concept validation, offers a versatile route toward the development of high-performance, low-cost electrocatalytic materials and electrochemical sensing platforms for environmental monitoring, energy conversion, and related applications.

## Figures and Tables

**Table 1:** XRD crystallographic parameters of N-NFC-O indexed to NiFe_2_O_4_ inverse spinel (JCPDS #10-0325, space group Fd-3m): observed and reference 2*θ* values, d-spacings, and relative peak intensities.

hkl	2*θ* (JCPDS) *	2*θ* (Obs.)	D-Spacing (Å)	Relative Peak Intensities
(220)	30.10	30.30	2.95	Weak
(311)	35.50	35.72	2.51	Strongest
(222)	37.10	37.10	2.42	Very weak
(400)	43.10	43.82	2.06	Moderate
(422)	53.40	53.50	1.71	Weak
(511)	57.00	57.20	1.61	Moderate
(440)	62.60	62.86	1.48	Moderate

* Reference: JCPDS#10-0325, Fd-3m. Scherrer crystallite size: 7–10 nm.

**Table 2:** XPS survey-scan elemental composition of N-NFC-O: peak binding energies, full width at half maximum (FWHM), atomic percentages, and chemical assignments for C 1s, O 1s, N 1s, Fe 2p, and Ni 2p core levels.

Element	Peak BE (eV)	FWHM (eV)	Atomic%	Assignment
C 1s	284.79	1.43	84.51	Rgo+biomass carbon
O 1s	532.62	3.37	10.68	M–O, C=O, C–OH
N 1s	400.25	3.67	3.13	Urea-derived N-doping
Fe 2p	711.34	4.95	1.08	Fe^2+^/Fe^3+^in NiFe_2_O_4_
Ni 2p	856.04	2.94	0.60	Ni^2+^/Ni^3+^in NiFe_2_O_4_

**Table 3:** BET and BJH pore structure parameters comparing NFC-S and N-NFC-O: specific surface area, total pore volume, average pore diameter, micropore fraction, and isotherm classification.

Parameter	NFC-S (Stepwise)	N-NFC-O (One-Pot)	Enhancement
BET surface area (m^2^ g^−1^)	5.23	186.39	×35.6
Total pore volume (cm^3^ g^−1^)	n.m.	0.1564	-
Avg. Pore diameter (nm)	n.m.	3.36	-
BJH avg. Pore diam. (nm)	n.m.	5.35	-
Micropore fraction (%)	n.m.	70.1	-
Isotherm type	n.m.	Type IV+H3	Mesoporous

n.m. = not measured due to carbon-shell encapsulation of NFC-S.

**Table 4:** Electrochemical performance parameters of bare GCE, NFC-S/GCE, and N-NFC-O/GCE in 5 mM [Fe(CN)_6_]^3−^/^4−^ + 1 M KCl at 50 mV s^−1^: anodic/cathodic peak currents, peak current ratio, peak-to-peak separation, and charge transfer resistance.

Electrode	Material	I_pa_ (μA)	I_pc_ (μA)	I_pa_/I_p_c	ΔE_p_ (mV)	R_ct_ (Ω)
G1	Bare GCE	117.89	−117.32	1.00	71.8	1419.4
G2	NFC-S stepwise/GCE	106.35	−113.28	0.94	113.3	1586.7
G3	N-NFC-O one-pot/GCE	112.60	−113.54	0.99	72.3	1459.2

**Table 5:** Comparison of analytical performance of the N-NFC-O/GCE sensor with other reported electrochemical nitrite sensors.

Electrode Material	Linear Range (µM)	Limit of Detection (μM)	Reference
AuNP/Graphene-Chitosan	0.9–18.9	0.30	[61]
Carboxyl graphene/PPy/Chitosan	0.2–1000	0.02	[62]
AuNPs/CS/Ti3C2	0.5–3355	0.069	[63]
N-NFC-O/GCE	200–2000	92.5	This work

**Table 6:** Comparison of synthesis parameters between the stepwise-synthesized NFC-S and the one-pot optimized N-NFC-O composites, including pyrolysis conditions, precursor compositions.

Parameter	NFC-S (Stepwise, Control)	N-NFC-O (One-Pot Optimized)
Synthesis approach	Five-step sequential	One-pot, single vessel
Fe precursor	FeCl_3_·6H_2_O (135.2 mg, 0.5 mmol)	Fe(NO_3_)_3_·9H_2_O (404.0 mg, 1.0 mmol)
Ni(NO_3_)_2_·6H_2_O	145.4 mg (0.5 mmol)	145.4 mg (0.5 mmol)
Fe:Ni molar ratio	1:1 (equimolar)	2:1 (stoichiometric)
GO reduction	Chemical (Vc, 90 °C, 3 h)	Thermal (in situ, pyrolysis)
Citric acid	—	315 mg
Urea	—	250 mg
L-Ascorbic acid	200 + 50 mg	—
pH adjustment	NaOH to ~9.5	—
Cotton mass	1.0 g	0.40 g
Pyrolysis temp./time	500 °C/2 h	600 °C/1.5 h
Pyrolysis ramp	5 °C/min (single)	Three-stage
N-doping source	None	Urea (250 mg)
GO	50 mg	50 mg

## Data Availability

The original contributions presented in this study are included in the article and/or Supplementary Materials. Further inquiries can be directed to the corresponding author.

## Supplementary Materials

The following supporting information can be downloaded at: https://www.mdpi.com/article/10.3390/molecules31172954/s1, Figure S1. DPV voltammograms of N-NFC-O/GCE recorded within the linear detection range (0.2–2.0 mM NaNO_2_) in 0.1 M PBS (pH 6.0). Pulse amplitude: 50 mV; pulse width: 0.05 s; step potential: 4 mV.

## Abbreviations

The following abbreviations are used in this manuscript:

AC	Alternating current
BET	Brunauer–Emmett–Teller
BJH	Barrett–Joyner–Halenda
CV	Cyclic voltammetry
DI	Deionized
DPV	Differential pulse voltammetry
DSC	Differential scanning calorimetry
DTG	Derivative thermogravimetry
EDS	Energy-dispersive X-ray spectroscopy
EIS	Electrochemical impedance spectroscopy
FE-SEM	Field-emission scanning electron microscopy
FTIR	Fourier-transform infrared spectroscopy
FWHM	Full width at half maximum
GCE	Glassy carbon electrode
GO	Graphene oxide
HRTEM	High-resolution transmission electron microscopy
IUPAC	International Union of Pure and Applied Chemistry
JCPDS	Joint Committee on Powder Diffraction Standards
K-PBS	Potassium phosphate buffer solution
LOD	Limit of detection
NFC-S	NiFe_2_O_4_/rGO@C composite (stepwise-synthesized)
N-NFC-O	N-doped NiFe_2_O_4_/rGO@C composite (one-pot optimized)
R_ct	Charge transfer resistance
rGO	Reduced graphene oxide
RSD	Relative standard deviation
RT	Room temperature
SAED	Selected-area electron diffraction
sccm	Standard cubic centimeters per minute
SEM	Scanning electron microscopy
TEM	Transmission electron microscopy
TGA	Thermogravimetric analysis
XPS	X-ray photoelectron spectroscopy
XRD	X-ray diffraction
ΔE_p	Peak-to-peak separation
ν	Scan rate
