# Supplementary material for: Overcoming Carbon-Shell Passivation in Biomass-Templated NiFe2O4/rGO@C Composites via a Urea-Assisted One-Pot Optimization Strategy for Enhanced Electrochemical Nitrite Sensing
Source: Molecules. 2026 Aug 23;31(17):2954. doi: 10.3390/molecules31172954 (PMC13567358; doi:10.3390/molecules31172954)

Supplementary Materials

Overcoming Carbon-Shell Passivation in Biomass-Templated $NiFe_2O_4$/rGO@C Composites via a Urea-Assisted One-Pot Optimization Strategy for Enhanced Electrochemical Nitrite Sensing

HanXu Liu, Khi Khim Beh, Jia Li, Wanling Lin, Chunhua Liu and Mohamad Adzhar Md Zawawi *

Figure S1. DPV voltammograms of N-NFC-O/GCE recorded within the linear detection range (0.2–2.0 mM $NaNO_2$) in 0.1 M PBS (pH 6.0). Pulse amplitude: 50 mV; pulse width: 0.05 s; step potential: 4 mV.

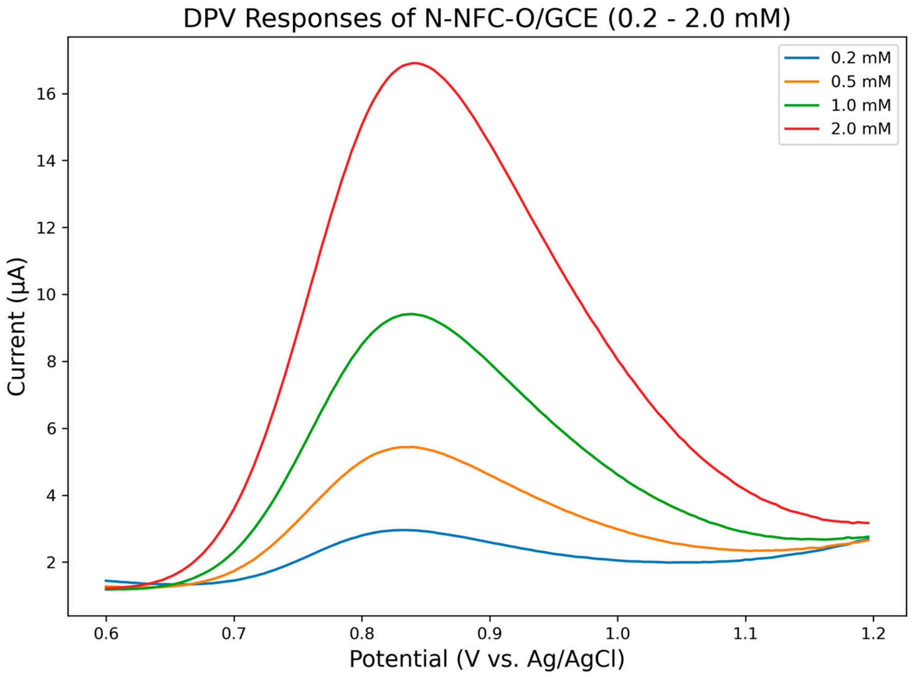

Supplement: Supplementary file 1 [file molecules-31-02954-s001.zip › molecules-4479791-supplementary.pdf]
